# Expert Consensus on Ocular Safety During Laser Procedures: A Practical Guide to Eye Safety by a Panel of Dermatologist Laser Surgeons and Ophthalmologists

**DOI:** 10.1111/jocd.70339

**Published:** 2025-07-10

**Authors:** Katarina Kesty, Chelsea Kesty, Jenna Benson, Kenneth Kesty, Cynthia Kesty

**Affiliations:** ^1^ St. Petersburg Skin and Laser St. Petersburg Florida USA; ^2^ Conway Ophthalmology Associates Conway South Carolina USA

**Keywords:** blepharoplasty, carbon dioxide laser, erbium, laser resurfacing, laser surgery, lasers, ocular complications, patient safety

## Abstract

**Objectives:**

This study aims to provide laser surgeons with guidelines to minimize ocular risks during laser procedures near the eyes. Complications such as corneal burns, infection, dry eye, cataracts, and blindness underscore the need for comprehensive safety protocols, incorporating ocular anatomy, patient‐specific factors, and proper use of protective equipment.

**Materials and Methods:**

A panel of Board‐Certified Dermatologists and Ophthalmologists with subspecialties in corneal and oculoplastic surgery was convened to develop recommendations. The panel addressed preoperative evaluations, intraoperative safety measures, and postoperative care to optimize patient outcomes.

**Results:**

Preoperative evaluations must include detailed medical and ocular histories, physical examinations, and screenings for contraindications like corneal disease or eyelid laxity. Eye shields, made of stainless steel and appropriately lubricated, are mandatory during laser procedures around the eye. Intraoperative monitoring ensures shield stability and patient comfort, with immediate cessation of the procedure if ocular pain or vision changes occur. Postoperative care includes sterile eye drops, follow‐ups to monitor ocular health, and patient education on recognizing complications.

**Conclusion:**

Rigorous protocols for laser procedures near the eyes ensure patient safety and minimize complications. Adherence to these recommendations allows for safe and effective outcomes in periocular laser treatments.

## 1 Objectives

Almost 80% of consumers are looking for a solution to the lines and wrinkles around the eye [1]. With the increasing popularity of eye rejuvenation using lasers and the concurrent rise in reported complications, we have assembled a panel of experts, including Dermatologist Laser Surgeons and Ophthalmologists from various subspecialties, to provide guidance to laser surgeons on minimizing ocular risks. The risks associated with laser procedures near the eyes are significant and can include corneal burns, ectropion, infection, dry eye, lamellar keratitis, cataracts, temporary or permanent blindness, and corneal or scleral scarring [2, 3, 4, 5, 6]. Addressing these risks requires a comprehensive understanding of ocular anatomy, patient‐specific factors, and the proper use of protective equipment.

## 2 Methods

We assembled a panel of 5 physicians, including Board Certified Dermatologists and Board Certified Ophthalmologists, to discuss the ocular risks of laser surgery and to discuss how to maximize safety during lasers. This panel will offer practical advice on patient care before, during, and after laser surgery, ensuring the highest level of safety. Our expert panel includes a Double Board‐Certified Dermatologist with board certifications in Dermatology and Mohs Micrographic Surgery and a fellowship in lasers and cosmetics from the American Society of Dermatologic Surgery, another Double Board‐Certified Dermatologist with board certifications in Dermatology and Mohs Micrographic Surgery, a Board‐Certified Ophthalmologist, a Board‐Certified Ophthalmologist with a fellowship in Cornea and anterior segment surgery, and a Board‐Certified Ophthalmologist with a fellowship in Oculoplastic Surgery.

## 3 Results

Laser resurfacing is a complex procedure that requires a skilled and experienced laser surgeon to balance patient safety and complication avoidance with optimal results. This requires choosing the right laser and wavelength or combination of lasers, and the optimal settings for the patient [6, 7]. Lasers around the eye are an especially delicate procedure, as we have another organ to consider during the laser procedure [8, 9]. Standards for laser resurfacing of the face have been discussed elsewhere, and this review will be focusing specifically on special considerations when doing lasers around the eye [10]. All patient safety considerations and standards of care should be followed when resurfacing the face, as well as additional considerations, discussed here, when performing laser close to the eye [11, 12, 13, 14, 15].

## 4 Preoperative Considerations

Before performing laser procedures, it is critical to conduct a thorough preoperative evaluation. This includes assessing the patient's medical history, such as a family history of glaucoma, systemic conditions like hypertension, diabetes, or heart disease, smoking or alcohol use, and medications such as oral isotretinoin or blood thinners. Specific ocular history should also be reviewed, including any contraindications to eye shield use and prior difficulties with eye shield application. Known conditions such as corneal edema, which weakens the epithelial barrier and is a major contraindication, should be discussed prior to laser surgery. For instance, a 2003 study highlights that screening for corneal edema can significantly reduce the risk of laser‐induced burns [2]. A history of glaucoma in the patient should be discussed, as the type of glaucoma may be a contraindication to the insertion of eye shields. Any history of orbital surgery, retraction, ectropion, or periocular surgery must be documented.

An intake form tailored to the specific procedure, whether laser blepharoplasty or resurfacing, should be completed. Screening for allergies to metals, eye drops, preservatives, or numbing medications is essential. Prior surgical history, such as cataracts, severe dry eye, lid lifts, facelifts, skin cancer surgery near the eyes, or trauma to the periocular area, should also be noted. Measurements of the eyelids should be taken preoperatively and on the day of blepharoplasty surgery using a protractor for accuracy. A thorough physical exam should also be performed for any laser surgery around the eye to check for ectropion, tendon laxity, and dry eye with a Schirmer test, as necessary. Documentation of true ptosis involving the lid margin and its proximity to the cornea is crucial. If the patient has severe eyelid laxity, eye symptoms, or significant ptosis, a preoperative ophthalmology exam is recommended, though a yearly exam is generally advised for all patients. An ophthalmologist should be consulted if the laser surgeon is unfamiliar with the ocular history of the patient, or it is unclear whether laser surgery around the eye is safe for the patient.

Eyelashes are a cosmetic concern for patients and should be discussed prior to laser around the eye. Depending on the laser procedure performed, the risk of singeing or damage to the eyelashes should be discussed. A 2000 study reported a 5%–10% incidence of temporary eyelash loss with CO_2_ lasers, underscoring the need for patient education [3]. Lash extensions should be removed prior to the laser procedure to prevent fire or damage to the extensions. Depending on the wavelength used for the laser, rarely eyelashes may be affected and not regrow.

Preoperative checklists should be performed for every procedure. This has been shown to decrease errors in medicine in general, and has been beneficial for patient flow and safety. The checklist should include a thorough list of necessary steps to prepare for the laser surgery. Tailoring the checklist to the procedure, laser surgeon preference, room setup, and clinic flow is necessary. Preoperative checklists for lasers around the eye should include the features listed in Table 1.

**TABLE 1:** Preoperative checklist. Example preoperative checklist for laser surgery around the eye including a column for the initials of the assistant or physician that completed that step.

Checklist item	Initials
Consent signed	
Patient preoperative pictures taken	
Review of post‐care procedures	
Schedule a postoperative appointment	
Allergies?	
Postoperative medicines sent to pharmacy	
Discussion of procedure and opportunity for patient to ask questions	
Thorough cleaning of face/eyelids	
Tray and laser suite room set‐up including specifics for each surgeon's according to their preferences	
Post‐procedure operations including giving patients and/or their caregivers instructions, including the phone number to call with any questions/concerns	

Written and verbal consent must include a discussion of the risks of blindness and other ocular complications. Specific postoperative instructions should be written out for each patient and given to them prior to the laser. These should include the phone number of whom they should call after‐hours if they have a question or concern. Postoperative instructions for laser around the eye should also include specific instructions regarding when a patient should proceed directly to the emergency room, including severe eye pain, bulging or protrusion of the eyeball, or any changes in vision.

## 5 Intraoperative Considerations

**FIGURE 1:**
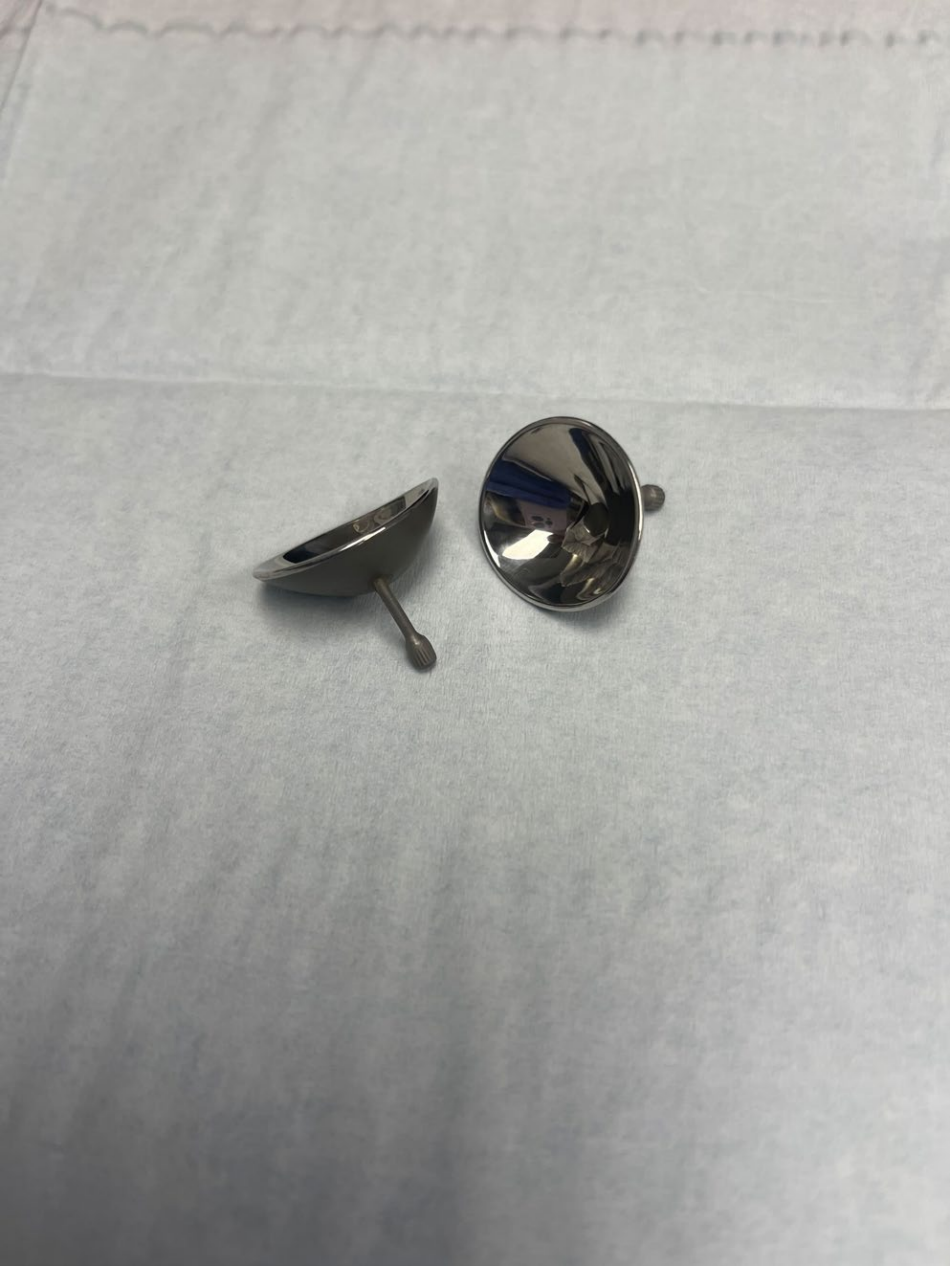
Metal eye shields used during laser surgery around the eye.

**FIGURE 2:**
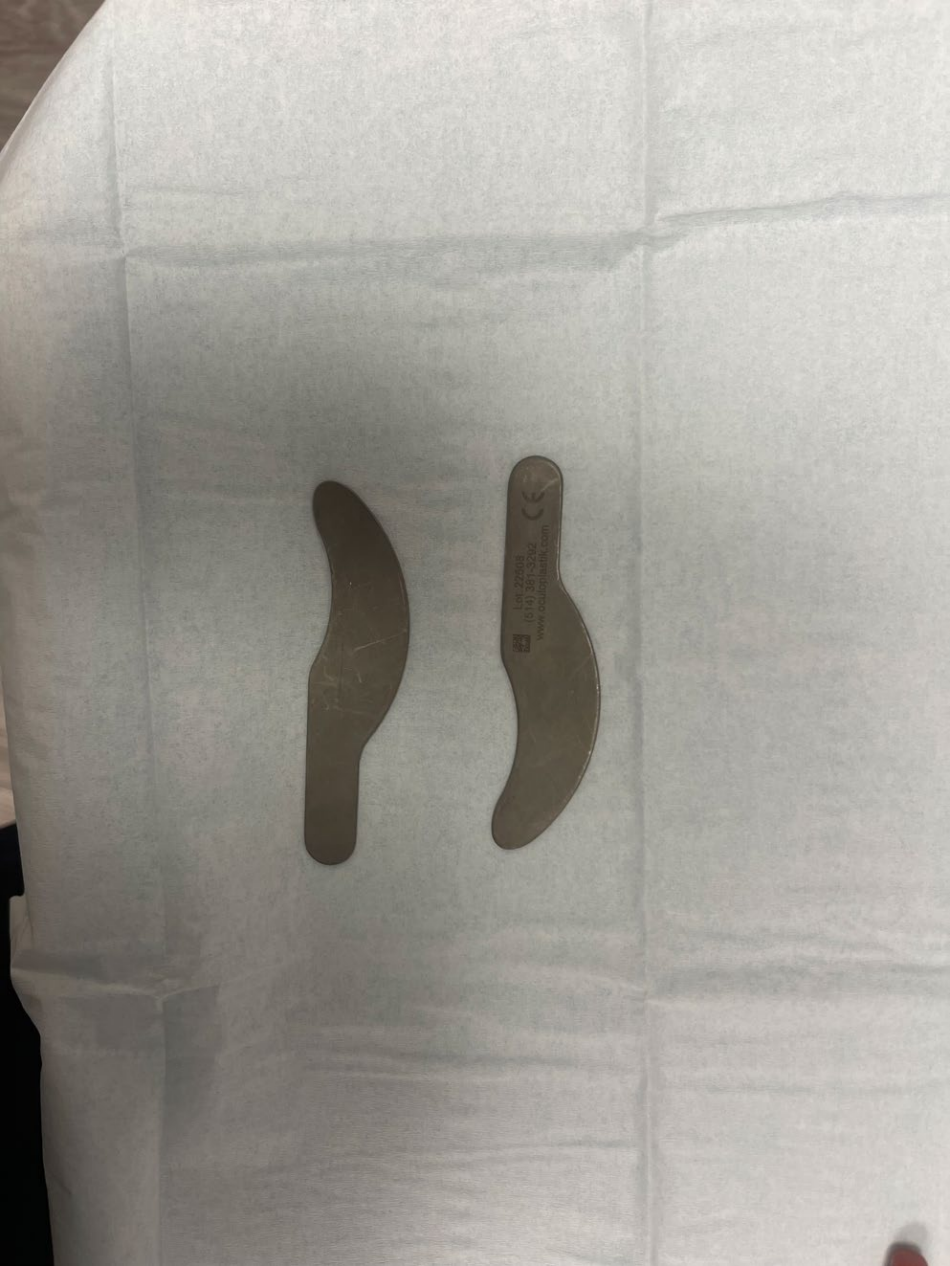
Metal Sutcliffe oculoplastic curved shield used during laser surgery around the eye.

Eye shields are mandatory for any laser procedure within the bony orbit, as all laser wavelengths pose some danger to ocular structures (Figure 1). During surgery, the use of proper eye shields is paramount. Shields should be made of stainless steel, 1 mm thick, autoclavable, and are available in small, medium, and large sizes to fit patients appropriately. Fader and Rainer (2000) found that properly fitted stainless steel eye shields reduce the risk of ocular injuries to near zero [12]. Shields with a “post” are preferred to ensure correct placement throughout the laser surgery as they can move within the eye. For blepharoplasty, a special metal exterior curved or flat Sutcliffe laser shield may be used to protect surrounding tissues when specificity or incision with the laser is required (Figure 2). Prior to sterilization, shields should be cleaned with a soft toothbrush. Shields should not be placed in an ultrasonic cleaner, as the solution can irritate the cornea when used for future patients. If placed in the ultrasonic cleaner, the shields will appear cloudy or hazy on the interior surface after sterilization. They should be opened and scrubbed gently with a soft toothbrush and mild cleanser to remove the buildup, then re‐sterilized for future use.

Sterile numbing eye drops, such as tetracaine hydrochloride ophthalmic solution 0.5%, should be used prior to shield insertion. These drops can cause a stinging sensation for the patient. LI et al. (2018) note that excessive use of tetracaine can increase postoperative dryness by 5%–10% [4]. The laser surgeon may consider flushing with 2–3 drops of normal preservative‐free tears after tetracaine drop insertion if necessary. The numbing tetracaine drops should be administered cautiously, as excessive use can increase postoperative dryness. Sterile, preservative‐free, lubricant eye gel containing hypromellose, polyethylene glycol, or propylene glycol should be generously applied to the shields or directly to the eyes to protect the cornea during metal shield insertion and during use of the shields. Excess lubrication that spills onto the eyelid skin should be removed with gauze to prevent interference with the laser. During the laser surgery, the laser surgeon should be monitoring for lubrication or other materials on the eyelids to prevent the laser having decreased or increased effectiveness. Additionally, the patient's head should be stabilized by an assistant or a device to prevent movement during the procedure. Patient movement during laser surgery can pose a safety risk, so assistants should be present and holding the head if necessary to prevent movement (involuntary or voluntary) by the patient.

Insertion of the shields requires careful technique (Video S1). The laser surgeon should hold the metal post and instruct the patient to look toward their feet during insertion (Video S1). The laser surgeon can hold the lids open with one hand and insert the shield with the other. Throughout the procedure, shields must be monitored to ensure they remain intact and correctly positioned in order to protect the eye from the laser. When wiping the skin clean in between passes of the laser, the laser surgeon must ensure that the shields do not get moved and scratch the cornea with a fast movement. The laser surgeon must also consider the pressure applied to the eye itself. The eye can tolerate moderate pressure, but careful monitoring is necessary. Cooling mechanisms, including cold air and ice packs, should be employed to monitor and manage the skin's temperature during the procedure, along with careful tracking of the number of laser passes and settings. A high number of passes or improper settings can overheat the eyelid skin and cause scarring of the skin or heat transfer to the cornea or other eye structures with ocular complications. The laser surgeon should be constantly monitoring the eye area, including manually feeling the temperature of the skin, to ensure safety. Emerging technologies such as real‐time thermal monitoring devices can further enhance safety by alerting surgeons to excessive skin temperatures, potentially reducing thermal injury risk.

If the patient reports heat or eye pain, the procedure should be stopped immediately, shields removed, and the patient's vision reassessed. Any significant concerns, such as retrobulbar hemorrhage or vision changes, should prompt referral to an emergency department.

The tray for a laser blepharoplasty should be sterile. For laser resurfacing around the eye, a clean tray with sterile supplies is adequate, but a sterile tray is an option. The gauze and drapes used during laser surgery pose a fire risk and can catch fire if the laser hits them. If a fire starts, follow your clinic's fire procedures. Always have the laser in the “Standby” mode until the laser surgeon is ready to start the case. Only the laser surgeon should switch the laser into “ready” mode, and only after the checklist is completed and the patient and all assistants have proper eye protection in place. Assistants should always be wearing appropriate laser eye protection when they are in the operating room.

## 6 Postoperative Care

Postoperatively, patients should be monitored for ocular symptoms. A follow‐up visit on the first postoperative day is critical to evaluate vision and examine the eyes for any signs of infection or dryness. Alharbi et al. (2023) report a 20%–30% reduction in complications with first‐day follow up [6]. Patients should be provided with sterile, preservative‐free eye drops, such as hydroxypropyl methylcellulose gel, and instructed on their proper use to prevent contamination. These drops can be useful to make patients comfortable after laser surgery around the eye and can be used liberally while the eyelid skin is healing. Petrolatum jelly may be used as a post‐laser salve to help with healing, but laser surgeons must ensure that it is pure petroleum jelly and does not have any added fragrances or other allergens. Petroleum jelly is safe to use close to the eye and may help with post‐laser dry eye. Patients must also be educated on recognizing signs of infection, including redness, pain, swelling, visual changes, and true pus, as opposed to postoperative exudate. Any suspicions of infection or irritation require immediate evaluation. Hamilton and Kao (2020) found a 2%–5% infection rate in non‐compliant patients, highlighting the importance of patient education [5].

Patients should be given written postoperative instructions, including when to seek medical attention, and provided with the contact information of their surgeon and assistants. Tear film recovery typically takes about a month and can be mitigated with artificial tears and other strategies to reduce dryness. Digital and artificial‐intelligence‐based follow‐up tools, such as patient apps for symptom reporting, are an innovative approach to improve compliance and early detection of complications.

## 7 Conclusion

In conclusion, laser surgeons performing procedures near the eyes must adopt rigorous preoperative, intraoperative, and postoperative protocols to safeguard their patients ocular health. Adherence to these precautions ensures the highest standard of care and minimizes the risk of complications, allowing for safe and successful outcomes.

## Author Contributions

K.K. and C.K. conceived the study and wrote and revised the manuscript. All authors have reviewed and approved the article for submission.

## Conflicts of Interest

The authors declare no conflicts of interest.

## Supporting information


**Video S1.** Video of eye shield insertion by Dr. Kat Kesty.

## Data Availability

Research data are not shared.
